# Phase II trial of nilotinib in PDGFR-alpha-enriched recurrent high-grade gliomas

**DOI:** 10.1093/noajnl/vdaf150

**Published:** 2025-07-10

**Authors:** David Piccioni, Tiffany M Juarez, Sneha L Kesari, Lara Rose, Natsuko Nomura, Santosh Kesari

**Affiliations:** Neuro-Oncology Program, Moores Cancer Center, UC San Diego, La Jolla, CA, USA; Department of Translational Neurosciences, Pacific Neuroscience Institute, Saint John’s Cancer Institute at Providence Saint John’s Health Center, Santa Monica, CA, USA; Neuro-Oncology Program, Moores Cancer Center, UC San Diego, La Jolla, CA, USA; Marshall College, UC San Diego, San Diego, CA, USA; Neuro-Oncology Program, Moores Cancer Center, UC San Diego, La Jolla, CA, USA; Department of Translational Neurosciences, Pacific Neuroscience Institute, Saint John’s Cancer Institute at Providence Saint John’s Health Center, Santa Monica, CA, USA; Neuro-Oncology Program, Moores Cancer Center, UC San Diego, La Jolla, CA, USA; Department of Translational Neurosciences, Pacific Neuroscience Institute, Saint John’s Cancer Institute at Providence Saint John’s Health Center, Santa Monica, CA, USA; Neuro-Oncology Program, Moores Cancer Center, UC San Diego, La Jolla, CA, USA

**Keywords:** glioblastoma, *MGMT*, nilotinib, PDGFRA

## Abstract

**Background:**

This phase II clinical trial evaluated the safety and efficacy of nilotinib in patients with recurrent, platelet-derived growth factor receptor alpha (PDGFRA)-enriched high-grade gliomas.

**Methods:**

Thirty-four adult patients with PDGFRA-enriched recurrent high-grade gliomas were enrolled. Study treatment consisted of nilotinib 400 mg administered twice daily in 28-day cycles. Safety and clinical activity were evaluated.

**Results:**

Median lines of prior therapy were 2 (range 1–7) and 9 of 34 (26%) patients received prior bevacizumab. Four patients had *PDGFRA* gene amplification, and 30 had PDGFRA overexpression by immunohistochemistry. Overall, nilotinib was well tolerated. The most common treatment-related toxicities were increased ALT, joint pain, and hyponatremia. No treatment-related grade 4 or 5 adverse events occurred. The best response was stable disease (SD) for 8 patients and complete response (CR) for one patient with glioblastoma. The median PFS was 1.45 months (95% CI 0.986–2.07) and the median OS was 6.6 months (95% CI 4.9–18.3). The patient with a CR was an *MGMT*-unmethylated GBM with PDGFRA overexpression by IHC, and maintained a durable response for over 5 years.

**Conclusion:**

Nilotinib was well tolerated with limited benefit in this enriched population of patients. Further studies are warranted to determine the clinical benefit in patients in earlier lines of treatment.

**Trial registration number:** NCT01140568, registered 08 June 2010.

**Key Points**

- Nilotinib is safe in high-grade gliomas.
- Nilotinib had limited benefit in recurrent high-grade gliomas.

**Importance of the Study**

This study shows that nilotinib was safe but has limited activity in biomarker-selected recurrent high-grade gliomas. There were some patients who had exceptional responses, and there is a need to better understand the full genomic aberrations in gliomas and how that can affect response to targeted therapies. Furthermore, it is likely that combination therapies are needed to induce higher responses and overcome resistance mechanisms.

Despite standard treatment with combined chemo-radiotherapy, with or without tumor-treating fields, essentially all patients with glioblastoma (GBM) will experience disease progression, and response rates in recurrent disease are less than 20%. With a median survival under two years and limited treatment options,^1,2^ more effective regimens are clearly needed for patients with recurrent GBM. Genome-wide studies have demonstrated the heterogeneous nature of GBM and motivated efforts to subclassify GBM according to shared molecular aberrations to better guide targeted therapy.^3^

Platelet-derived growth factor (PDGF) and PDGF receptor (PDGFR) autocrine loops are implicated in the pathogenesis of one of the biologically distinct subgroups of GBM.^4,5^ Co-expression of PDGF A and B ligands, and the PDGF alpha receptor (PDGFRA), is common in most malignant gliomas, while the PDGF beta receptor is frequently expressed in glioma and endothelial cells.^6–9^ Activation of the PDGF/PDGFR signaling pathway can lead to cancer proliferation, metastasis, invasion, and angiogenesis.^10^ PDGFR is overexpressed in approximately 15% of GBMs^11^ and aberrant signaling is thought to initiate cellular transformation and contribute to the transformed phenotype.^12–14^ Accordingly, selecting patients with tumors overexpressing PDGFRA is one potential strategy to improve sensitivity to treatment with kinase inhibitors.

Nilotinib is a second-generation multi-kinase inhibitor approved as treatment for Philadelphia-chromosome-positive chronic myelogenous leukemia. In addition to inhibiting Bcr-Abl tyrosine kinase, it targets type III split domain tyrosine kinase family (c-kit, PDGFRA, FLT-3), thus inhibiting PDGFRA signaling.^15–17^ Preclinical data showed increased potency of nilotinib compared to first-generation imatinib in inhibiting the growth of human glioma cell lines and stem cells.^18^ These findings, together with evidence indicating less susceptibility to resistance via active cellular transporter pumps,^19^ we hypothesized that nilotinib might improve response in biomarker-enriched patients.

We therefore conducted an investigator-initiated, single-arm single-center phase II trial of nilotinib to evaluate the safety and antitumor activity in patients with PDGFR inhibitor-naive progressive or recurrent high-grade gliomas enriched for PDGFR-alpha pathway.

## Methods

### Patient Selection

Approval by the UC San Diego Institutional Review Board was obtained, and the study was conducted in accordance with the Declaration of Helsinki and the International Conference on Harmonization Good Clinical Practice Guidelines and registered on clinicaltrials.gov (NCT01140568). All patients provided written informed consent.

Eligible patients were ≥ 18 years with recurrent malignant gliomas, with either *PDGFRA* gene amplification or PDGFR-alpha overexpression by immunohistochemistry (IHC). The study initially allowed grade III and grade IV infiltrating gliomas (according to the World Health Organization (WHO) 2007 classification) but was subsequently amended to include only grade IV glioblastoma. Disease assessment according to WHO 2021 classification was retrospectively applied for publication (Supplementary **Table S1**). PDGFRA testing was performed at Clarient Diagnostics (currently NeoGenomics Laboratories) on baseline newly diagnosed tumor FFPE samples and reported as standard 0-3 + IHC staining criteria by pathologist (0: Negative; 1+: Weak staining; 2+: Moderate staining; 3+: Strong staining). The status of other critical biomarkers was also collected, including O-6-methylguanine-DNA methyltransferase (*MGMT*) gene and isocitrate dehydrogenase (*IDH*) based on clinical testing.

Patients had Karnofsky performance status ≥ 60%, a life expectancy of ≥ 3 months, and adequate bone marrow (absolute neutrophil count ≥ 1.5 × 10^9^/L, platelet count 100 × 10^9^/L, hemoglobin ≥ 9.0 g/dL), renal (serum creatinine ≤ 1.5 × institution’s upper limit of normal (ULN)), and liver (AST/SGOT and ALT/SGPT ≤ 2.5 × ULN, total bilirubin ≤ 1.5 × ULN, alkaline phosphatase ≤ 2.5 × ULN unless considered tumor-related) function. Patients were neurologically stable, defined as receiving either no, stable, or tapering doses of corticosteroids for at least 5 days prior to study drug initiation. Woman of child-bearing potential and men with partners of child-bearing potential agreed to use adequate contraception while on study. Patients were required to have recovered from acute toxic effects of prior therapy and not to have received investigational agents within 28 days of study entry; cytotoxic therapy within 28 days (42 days if nitrosourea, 23 days if temozolomide, 21 days if procarbazine, irinotecan or topotecan, or 14 days if vincristine); non-cytotoxic agents within 7 days, or surgery within 4 weeks. Patients were excluded if they had uncontrolled seizures within 5 days of study initiation; radiographic findings consistent with radiation necrosis; a history of allergic reactions attributed to compounds of similar chemical or biologic composition to nilotinib; a severe or uncontrolled concurrent medical disorder; impaired cardiac function; were on enzyme-inducing anti-epileptic drugs, known substrates of CYP2D6, warfarin, or proton pump inhibitors; or were pregnant or nursing females.

### Study Design

This was an open-label, single-center, phase II study to evaluate the efficacy and safety of daily oral nilotinib. A Simon’s two-stage design was used to test the null hypothesis H0 p ≤ 10% against the alternative hypothesis H1: p > 10%, where p is the tumor response rate at 2 months. Two of the first 11 patients were required to show stable disease (SD), partial response (PR), or complete response (CR) at the first assessment, before proceeding with full enrollment. The primary end point was progression-free survival at 6 months (PFS6). Secondary end points were safety, overall survival (OS), and objective response rate (ORR).

### Treatment Plan and Assessments

Nilotinib was administered at a dose of 400 mg orally by mouth twice daily, in 28-day cycles. This dose was chosen based on the highest dose approved for patients with resistant or intolerant chronic myeloid leukemia. Dose reductions were allowed for toxicity or tolerability. Toxicities were assessed according to the National Cancer Institute Common Toxicity Criteria for Adverse Events (NCI-CTCAE) version 4.03. A physical exam, neurological exam, and Karnofsky Performance Status (KPS) assessment were done prior to starting each cycle. A complete blood count with differential and complete metabolic panel was performed every two weeks. Tumors were imaged by MRI after every two treatment cycles, or earlier if clinically indicated, and response was assessed according to MacDonald criteria.

## Results

### Patient Characteristics

From February 2011 to August 2016, 34 patients were enrolled into the study and treated with nilotinib. Characteristics of the patients at baseline are summarized in **Table 1** and an individual listing is provided in Supplementary **Table S1**. Thirty-four patients were treated (24 GBM, 2 Grade 3 gliomas, and 8 Grade 4 IDH-mutant astrocytoma). 26 were male and 8 were female. Median age was 55.5 (range 22–78 years). Four patients had *PDGFRA* amplification, and 30 had PDGFRA overexpression by IHC. Median lines of prior therapy were 2 (range 1–7). Nine of 34 patients received prior bevacizumab.

**Table 1.** Patient Demographics and Clinical Characteristics of All Treated Patients (*N* = 34)

Characteristic	No. of patients	%
**Age, years**		
Median	55.5	
Range	22-78	
**Gender**		
Female	8	23.5%
Male	26	76.5%
**Racial Origin**		
Non-Hispanic White	29	85.3%
Hispanic	2	5.9%
Asian	3	8.8%
**Pathology**		
Glioblastoma IDHwt	24	70.6%
Grade 4 Astrocytoma IDHmut	8	23.5%
Grade 3 Glioma IDHwt	2	5.8%
***MGMT* Status**		
Unmethylated	13	38.2%
Methylated	6	17.6%
Unknown	15	44.1%
**Progression**		
1^st^	11	32.4%
2^nd^	10	29.4%
3+	13	38.2%
**Prior Bevacizumab**		
Yes	9	26.5%
No	25	73.5%
**PFS6**		
Yes	3	8.8%
No	31	91.2%

### Toxicity

All patients were assessed for toxicities (**Table 2**). Ten of 34 patients (29%) experienced treatment-related adverse events, and 6 patients had treatment-related grade 3 AEs. There was one grade 4 event, which was unrelated to the study drug and no grade 5 events. One patient discontinued study treatment after 5 days secondary to grade 3 pancreatitis (serious). Dose reductions occurred for elevated bilirubin (*n* = 1), elevated hepatic transaminases (*n* = 1), leg cramps (*n* = 1; serious), and joint pain (*n* = 2). All 5 were able to continue at the protocol-specified reduced dose.

**Table 2.** Number of Patients with Treatment-Related Adverse Events

	CTCAE Grade*			Total Patients (*n* = 34)	
	1	2	3	No.	%
**Investigations**					
Increased AST			1	**1**	**2.9%**
Increased Bilirubin			2	**2**	**5.9%**
Increased ALT	1	1	2	**4**	**11.8%**
Hypophosphatemia			1	**1**	**2.9%**
Hyponatremia	1		1	**2**	**5.9%**
Lipase			1	**1**	**2.9%**
Pneumonia			1	**1**	**2.9%**
**Musculoskeletal and Connective Tissue Disorders**					
Leg Cramping			1	**1**	**2.9%**
Joint Pain		2		**2**	**5.9%**
**Skin and Subcutaneous Tissue Disorders**					
Irritated Skin (Nipples)	1			**1**	**2.9%**

A.

B.

C.

### Antitumor Activity

**Figure 1.**
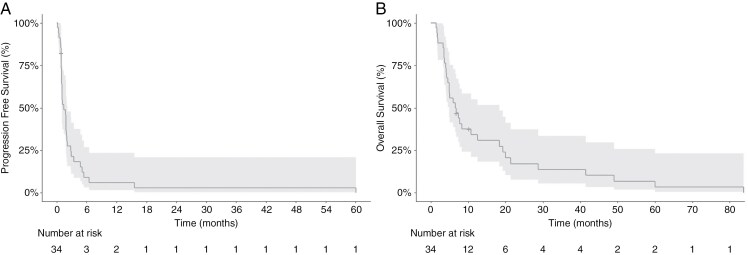
(a) PFS and (b) OS for all 34 patients. The shaded area represents 95% CI.

The PFS6 was 9% (3/34), and PFS12 was 6% (2/34). Median PFS was 1.45 months (95% CI 0.986-2.07), and the median OS was 6.6 months (95% CI 4.9-18.3) (**Figure 1**). The best response was stable disease (SD) for 8 patients and complete response (CR) for one patient. ORR was 1/34 patients (3%). The patient achieving a CR had *MGMT*-unmethylated GBM with PDGFRA overexpression by IHC, and maintained a durable response for over 5 years. One patient discontinued treatment at 15.6 months with SD, due to inability to return to the treating facility, but is still alive ongoing 12 years.

Considering that *PDGFRA* amplification and PDGFRA overexpression are biologically distinct entities, we performed an additional post-hoc analysis of patients stratified by PDGFRA status (Supplementary **Table S2**). Median PFS for the four patients with *PDGFRA* amplification was 2.05 months (95% CI 1.84-NA), and median OS was 11.6 months (95% CI 3.48-NA), with 1 SD. Median PFS for patients with PDGFR overexpression was 1.08 months (95% CI 0.95–2.83), and median OS was 6.62 months (95% CI 4.6–19.3), with 1 CR and 7 SD. While there is a trend for longer survival in amplified tumors, the sample size was too small to make any definite conclusions.

**Figure 2.**
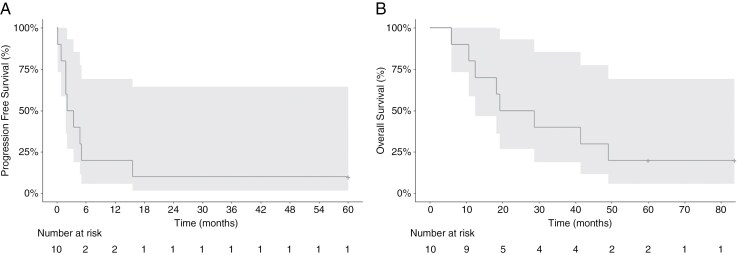
Post hoc analysis of (a) PFS and (b) OS for the 10 patients treated at first progression, with no prior exposure to bevacizumab. The shaded area represents 95% CI.

**Figure 3.**
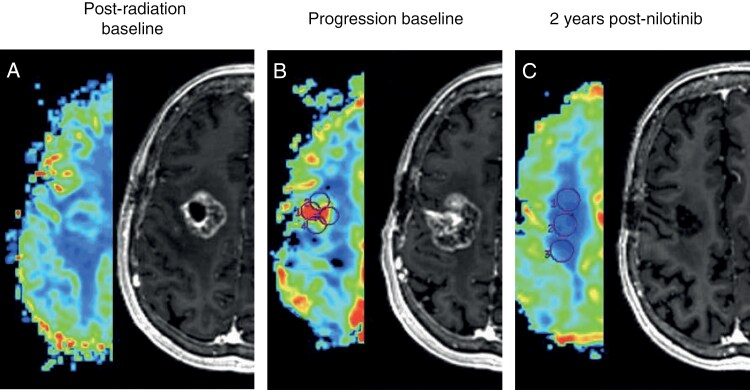
Complete response in an unmethylated, IDHwt glioblastoma following treatment with nilotinib. Perfusion and axial T1 post-contrast images pictured. (a) Post radiation baseline. (b) Recurrence with increased perfusion (circles). (c) Complete response at 2 years and maintained for over 5 years.

We performed a post hoc analysis of the patients who were treated at first recurrence and had not received any prior bevacizumab, as this is often the criteria for more recent trials in recurrent GBM. Ten patients met this criterion: 7 were male and 3 were female. Pathology was as follows: 8 GBM IDHwt, 1 Grade 4 astrocytoma IDHmut, and 1 Grade 3 glioma; *MGMT* was unmethylated in three, methylated in one, and unknown in 6. Two had *PDGFRA* amplification, and 8 had PDGFRA IHC overexpression. The median PFS for this group was 2.8 months (95% CI 1.84-NA), and the median OS was 24 months (95% CI 12.5-NA). The best response was 1 CR and 3 SD. Two of the 10 patients were alive at 5 years (**Figure 2**). The patient with the complete response had symptomatic and imaging progression, including increased perfusion by cerebral blood volume, prior to enrolling in the study. She then received treatment with nilotinib for 5 years with complete response on imaging (**Figure 3**) and symptomatic improvement.

## Discussion

Overall, nilotinib was safe and had limited activity in recurrent GBM enriched for PDGFRA, though one durable complete response and a few cases of long-term stable disease were observed. Although the study was not powered for PDGFRA status stratification, patients with *PDGFRA* amplification had improved overall survival compared to those with overexpression by IHC. Furthermore, a post hoc analysis showed improved overall survival in the subset of patients treated at first recurrence and without prior bevacizumab. This subgroup was also enriched for patients with *PDGFRA* amplification. Similarly, the RTOG 0627 phase 2 trial of dasatinib, another second-generation multi-kinase inhibitor, failed to improve outcomes in gliomas with PDGFRA overexpression by IHC.^20^ These findings highlight the need for further exploration of the genetic mechanisms driving aberrant PDGFRA pathway activation.

This study had several limitations, including biomarker grouping, a heavily pretreated patient population, and the use of outdated MacDonald criteria for response assessment. The study had a high burden of prior therapies (median two prior lines, up to seven) and prior bevacizumab use in about 25% of patients, which may have influenced outcomes. Not surprisingly, survival appeared more favorable when used earlier in the disease course. Evaluation of nilotinib in the pre-radiation or neoadjuvant setting in newly diagnosed disease could potentially circumvent the confounding effects of radiation necrosis and treatment-induced resistance to provide a more accurate assessment of drug activity.^21^ The limited clinical molecular profiling (in 2009 era) also restricted insights into mechanisms of resistance or response, and the study did not explore reasons for the heterogeneous duration of benefit observed among patients. Furthermore, given that some patients had prolonged response, including a CR, broader identification of biomarker-responsive patient cohorts in the future will improve PFS, OS, and response rates.

Further molecular characterization is warranted to identify other biomarkers that could be used to select patients who may benefit from nilotinib or PDGFRA inhibition. High-level receptor tyrosine kinase (RTK) amplifications, often associated with gene rearrangements, are maintained through positive selection and exhibit intratumoral heterogeneity due to unstable amplicon segregation and variable selection pressures. Subpopulations with distinct RTK genotypes can emerge through mechanisms such as polyclonal tumorigenesis, unequal segregation, or environmental influences. Analysis of the Cancer Genome Atlas (TCGA) reveals coamplification of RTKs, particularly EGFR and PDGFRA, in significant subpopulations of GBM cells, impacting therapeutic strategies.^22^ Distinct RTK-dependent subpopulations may contribute to poor responses to single-pathway inhibitors, advocating for alternative strategies like sequential therapies to minimize toxicity while targeting diverse tumor cell populations. Future studies should include phospho-PDGFRA-positive or *PDGFRA*-amplified tumors, and in earlier second-line or neoadjuvant settings without any prior bevacizumab. Drug delivery may also be an issue as the brain penetration of drugs into enhancing and non-enhancing tumor is complex. Newer techniques of liquid biopsies in blood and cerebrospinal fluid will also open up new opportunities to diagnose the molecular status of disease and track outcomes over time.

## Supplementary Material

vdaf150_suppl_Supplementary_Tables_S1-S2

## Data Availability

Patient clinical data generated, which is de-identified in this study, is available upon reasonable request from DP. The genetic sequencing data as part of the standard of care is not publicly available due to patient privacy requirements. Derived data supporting the findings of this study are available from DP upon reasonable request.
