# Supplementary material for: Phase II trial of nilotinib in PDGFR-alpha-enriched recurrent high-grade gliomas
Source: Neurooncol Adv. 2025 Jul 10;7(1):vdaf150. doi: 10.1093/noajnl/vdaf150 (PMC12288025; doi:10.1093/noajnl/vdaf150)
Supplement: vdaf150_suppl_Supplementary_Tables_S1-S2 [file vdaf150_suppl_supplementary_tables_s1-s2.docx]

# Table S1. Individual listing of clinical trial participants.

| **ID** | **Age** | **Sex** | **Race** | **KPS** | **2007 WHO**  **Diagnosis** | **2021 WHO Diagnosis** | **IDH1 Status** | **Progression No.** | **MGMT Methylation** | **PDGFRA amp or % stain** | **PDGFRA IHC** | **Bevacizumab Usage** | **Best Response** |
| --- | --- | --- | --- | --- | --- | --- | --- | --- | --- | --- | --- | --- | --- |
| P01 | 52 | M | Asian | 90 | AA | G3 glioma | n.d. | 1st | n.d. | amplified | n.d. | None | PD |
| P02 | 45 | M | White | 80 | GBM | GBM | n.d. | 3rd | n.d. | amplified | n.d. | Prior | PD |
| P03 | 36 | M | White | 90 | GBM | GBM | n.d. | 2nd | n.d. | 70 | 1+ | After study | PD |
| P04 | 60 | F | White | 70 | GBM | GBM | n.d. | 3rd | n.d. | 70 | 1+ | After study | PD |
| P05 | 65 | M | White | 70 | GBM | GBM | n.d. | 3rd | n.d. | 80 | 1+ | Prior | PD |
| P06 | 46 | M | White | 60 | GBM | GBM | n.d. | 2nd | n.d. | m | m | Prior | SD |
| P07 | 72 | M | White | 70 | GBM | GBM | n.d. | 1st | n.d. | amplified | n.d. | None | SD |
| P08 | 52 | M | White | 80 | GBM | GBM | wt | 1st | unmethylated | 60 | 1+ | After study | PD |
| P09 | 65 | M | White | 80 | IDHwt G3 AO | G3 glioma | wt | 2nd | unmethylated | 80 | 2+ | After study | PD |
| P10 | 70 | M | White | 70 | GBM | GBM | wt | 1st | unmethylated | 50 | 1+ | None | CR |
| P11 | 31 | M | Asian | 80 | GBM | GBM | wt | 5th | n.d. | 100 | 2+ | After study | PD |
| P12 | 62 | M | White | 90 | GBM | GBM | wt | 3rd | methylated | 70 | 1+ | Prior | PD |
| P13 | 45 | F | White | 80 | GBM | GBM | wt | 1st | unmethylated | 100 | 3+ | After study | PD |
| P14 | 54 | M | White | 90 | GBM | GBM | wt | 1st | n.d. | 50 | 1+ | After study | PD |
| P15 | 78 | M | White | 90 | GBM | GBM | wt | 1st | n.d. | 100 | 3+ | After study | SD |
| P16 | 66 | F | White | 80 | GBM | GBM | wt | 3rd | methylated | 70 | 1+ | After study | PD |
| P17 | 74 | M | White | 100 | GBM | GBM | mut | 1st | n.d. | 99 | 2+ | After study | SD |
| P18 | 54 | M | White | 90 | GBM | GBM | wt | 5th | unmethylated | amplified | n.d. | Prior | PD |
| P19 | 70 | F | White | 70 | DA IDHmut then GBM | G4 IDHmut astrocytoma | mut | 2nd | methylated | 100 | 3+ | After study | PD |
| P20 | 53 | M | Hispanic/ Latino | 90 | GBM | GBM | wt | 2nd | unmethylated | 70 | 2+ | Prior | PD |
| P21 | 59 | M | White | 70 | GBM | GBM | wt | 2nd | n.d. | 99 | 3+ | None | PD |
| P22 | 48 | M | White | 80 | GBM | GBM | wt | 2nd | methylated | 100 | 3+ | After study | SD |
| P23 | 41 | F | White | 60 | GBM | GBM | wt | 1st | methylated | 100 | 3+ | None | PD |
| P24 | 35 | M | Hispanic/ Latino | 80 | GBM | GBM | wt | 2nd | unmethylated | 100 | 3+ | None | PD |
| P25 | 57 | M | White | 80 | GBM | GBM | wt | 1st | n.d. | 100 | 3+ | None | SD |
| P26 | 27 | M | White | 60 | GBM | GBM | wt | 1st | unmethylated | 100 | 3+ | Prior | SD |
| P27 | 22 | M | White | 70 | GBM | GBM | n.d. | 3rd | unmethylated | m | m | Prior | PD |
| P28 | 70 | M | White | 60 | GBM | GBM | wt | 7th | unmethylated | 90 | 2+ | Prior | PD |
| P29 | 52 | M | Asian | 90 | GBM | GBM | wt | 3rd | unmethylated | 100 | 2+ | Prior | PD |
| P30 | 45 | M | White | 80 | GBM | GBM | wt | 2nd | unmethylated | 100 | 2+ | After study | PD |
| P31 | 36 | M | White | 70 | GBM | GBM | n.d. | 2nd | n.d. | 99 | 3+ | After study | SD |
| P32 | 60 | F | White | 80 | GBM | GBM | wt | 3rd | methylated | 99 | 3+ | After study | PD |
| P33 | 65 | M | White | 70 | DAwt then GBM | GBM | wt | 5th | unmethylated | m | m | None | PD |
| P34 | 46 | M | White | 90 | GBM | GBM | wt | 3rd | n.d. | 100 | 3+ | None | PD |

Abbreviations: AA = anaplastic astrocytoma; GBM = glioblastoma; G3 = Grade 3; AO = anaplastic oligodendroglioma; DA = diffuse astrocytoma; mut = mutated; n.d. = not done; wt = wildtype; PD = progressive disease; SD = stable disease; CR = complete response; m = positive at enrollment but info missing from database; (0: Negative; 1+: Weak staining; 2+: Moderate staining; 3+: Strong staining).

**Table S2. Efficacy outcomes by PDGFRA status.**

|  | ***PDFRA* amplification**  **(N=4)** | **PDGFRA overexpression**  **(N=30)** |
| --- | --- | --- |
| **Response** | 1 SD, 3 PD | 1 CR, 7 SD, 22 PD |
| **Response Rate** | 0% | 3% |
| **Disease Control** | 25% | 27% |
| **Median PFS, months (95% CI)** | 2.05 (1.84-NA) | 1.08 (0.95-2.83) |
| **Median OS, months (95% CI)** | 11.6 (3.48-NA) | 6.62 (4.6-19.3) |

Abbreviations: PFS = progression-free survival, OS = overall survival, CI = confidence interval, SD = stable disease, PD = progressive disease, CR = complete response..
